# Effect of gold nanoparticles on the structure and neuroprotective function of protein *L*-isoaspartyl methyltransferase (PIMT)

**DOI:** 10.1038/s41598-021-93752-1

**Published:** 2021-07-12

**Authors:** Tanaya Chatterjee, Gaurav Das, Surajit Ghosh, Pinak Chakrabarti

**Affiliations:** 1 Department of Biochemistry, Bose Institute, P1/12 CIT Scheme VIIM, Kolkata, 700054 India; 2 Organic and Medicinal Chemistry Division, CSIR-Indian Institute of Chemical Biology, 4, Raja S. C. Mullick Road, Jadavpur, Kolkata, 700032 India; 3 Academy of Scientific and Innovative Research (AcSIR), Ghaziabad, 201002 India; 4 Structural Biology and Bioinformatics Division, CSIR-Indian Institute of Chemical Biology, 4, Raja S. C. Mullick Road, Jadavpur, Kolkata, 700032 India; 5 Present Address: Department of Bioscience and Bioengineering, Indian Institute of Technology Jodhpur, Rajasthan, 342037 India

**Keywords:** Biochemistry, Biophysics, Neuroscience, Structural biology

## Abstract

Fibrillation of peptides and proteins is implicated in various neurodegenerative diseases and is a global concern. Aging leads to the formation of abnormal isoaspartate (isoAsp) residues from isomerization of normal aspartates in proteins, triggering fibril formation that leads to neurodegenerative diseases. Protein *L*-isoaspartyl methyltransferase (PIMT) is a repair enzyme which recognizes and converts altered isoAsp residues back to normal aspartate. Here we report the effect of gold nanoparticles (AuNPs) of different sizes on the structure and function of PIMT. Spherical AuNPs, viz*.* AuNS5, AuNS50 and AuNS100 (the number indicating the diameter in nm) stabilize PIMT, with AuNS100 exhibiting the best efficacy, as evident from various biophysical experiments. Isothermal titration calorimetry (ITC) revealed endothermic, but entropy driven mode of binding of PIMT with all the three AuNSs. Methyltransferase activity assay showed enhanced activity of PIMT in presence of all AuNSs, the maximum being with AuNS100. The efficacy of PIMT in presence of AuNS100 was further demonstrated by the reduction of fibrillation of Aβ42, the peptide that is implicated in Alzheimer’s disease. The enhancement of anti-fibrillation activity of PIMT with AuNS100 was confirmed from cell survival assay with PC12 derived neuronal cells against Aβ42 induced neurotoxicity.

## Introduction

Proteins undergo various post-translational modifications (PTM) due to aging which are implicated in various debilitating diseases^1–3^. Amongst various PTM, isomerization/racemization of normal aspartate (Asp)/asparagine (Asn) residues to isoasparate (isoAsp) residues is well known and occurs due to aging^4,5^. Protein *L*-isoaspartyl methyltransferase (PIMT) is a ubiquitous repair enzyme that recognizes these abnormal *L*-isoAsp residues as its substrate and converts them back to normal aspartate residues^6^. Formation and accumulation of these isoAsp can lead to various neurodegenerative diseases and the role of PIMT associated with neurodegeneration has been reported in literature^7–9^.

We have recently demonstrated the role of isoAsp in fibrillation using a pair of hexapeptides, one with isoAsp and another with Asp—only the former was found to form fibrils. PIMT was found to prevent the fibrillation of the isoAsp-containing peptide, as well as inducing the transition of β-sheet (observed in fibrils) to α-helix (seen in the physiologically active native form) in Aβ42 peptide that has been associated with Alzheimer’s disease^10^. Indeed ligands have been designed to bind and stabilize the 13–26 region of Aβ peptide in an α-helical conformation—this counteracts polymerization of the peptide into toxic assemblies, thereby providing a strategy for the development of specific inhibitors of Aβ polymerization^11^. PIMT may offer another approach for preventing the fibrillation by retaining the helical conformation of the peptide by converting isoAsp into normal Asp. It would then be useful to find ways and means to stabilize the structure of PIMT and increase its activity.

Nanoparticles, especially gold nanoparticles (AuNPs) are well known for biocompatibility and hence have wide applications in the field of biomedical research, ranging from cellular imaging to drug delivery^12–14^. Other unique features of AuNPs are their non-toxic and inert nature which makes them an excellent candidate for bioconjugation with various ligands^15,16^. Recent years have witnessed the use of AuNPs as promising therapeutic agents against fibrillation of peptides and proteins that are implicated in various neurodegenerative diseases^17–19^. In this context, considering the neuroprotective role of PIMT, we wanted to explore the role of spherical AuNPs of different sizes (AuNS5, AuNS50 and AuNS100, the number indicating the diameter of the nanospheres, AuNSs, in nm) in affecting the structure and function (notably the anti-fibrillation activity) of PIMT. The protein-NP interaction was studied using various biophysical techniques, such as far-UV CD, fluorescence spectroscopy, isothermal calorimetry (ITC) etc. PIMT exhibited enhanced methyltransferase activity in presence of AuNSs, the maximum efficacy being with AuNS100. The enhancement of anti-fibrillation activity of PIMT in presence of AuNSs of different sizes against Aβ42 was further confirmed from thioflavin T (ThT) fluorescence assay. To gain further insight and corroboration we used an Aβ42 induced neuronal model using PC12 derived neurons, and observed the enhancement of cellular survival against the Aβ42 induced toxicity on treatment with PIMT together with AuNSs that was higher than that with PIMT alone. To the best of our knowledge the structural/functional modification of this ubiquitous repair enzyme PIMT by AuNPs has not been reported in literature till date and is likely to have bearing on the therapeutic application against various neurodegenerative diseases.

## Results

### Changes in optical properties of AuNSs in presence of PIMT

The unique feature of AuNPs is their Surface Plasmon Resonance (SPR) bands which appear due to oscillation of conduction band electrons. For spherical AuNPs the SPR bands appear in the visible region. Fig. S1 shows the SPR bands of AuNSs alone and in conjugation with PIMT. For AuNS5 and AuNS50 the SPR absorption bands appeared at 526 nm and 535 nm, respectively, which on conjugation to PIMT, got shifted to higher wavelengths of 542 nm and 546 nm, respectively. To start with, the peak in the SPR spectrum of AuNS100 appeared at a higher value (569 nm) which also got red-shifted to 593 nm upon binding to PIMT. The shift in the wavelength maxima of AuNSs may be due to the change of local refractive index by the adsorption of PIMT^20^.

### Adsorption of PIMT on AuNSs using UV–vis spectroscopy

The adsorption of PIMT on AuNSs was carried out by measuring the light absorption at 280 nm with the increase in concentration of the protein, while keeping that of the nanoparticle constant. In the presence of AuNSs, the OD was found to increase monotonically with the concentration of PIMT (Fig. S2b). For the control experiment in the absence of AuNSs, the same trend of increasing OD was seen with increasing PIMT concentration (Fig. S2a). The difference in the OD for the PIMT-AuNSs conjugates from the OD of the free PIMT (*ΔA*) revealed the adsorption of protein to nanoparticles (Fig. S2). The adsorption of PIMT was found to be more for the smallest AuNS5, as compared to its larger counterparts, AuNS50 and AuNS100.

### Unfolding of PIMT and PIMT in presence of AuNSs using urea

**Figure 1:**
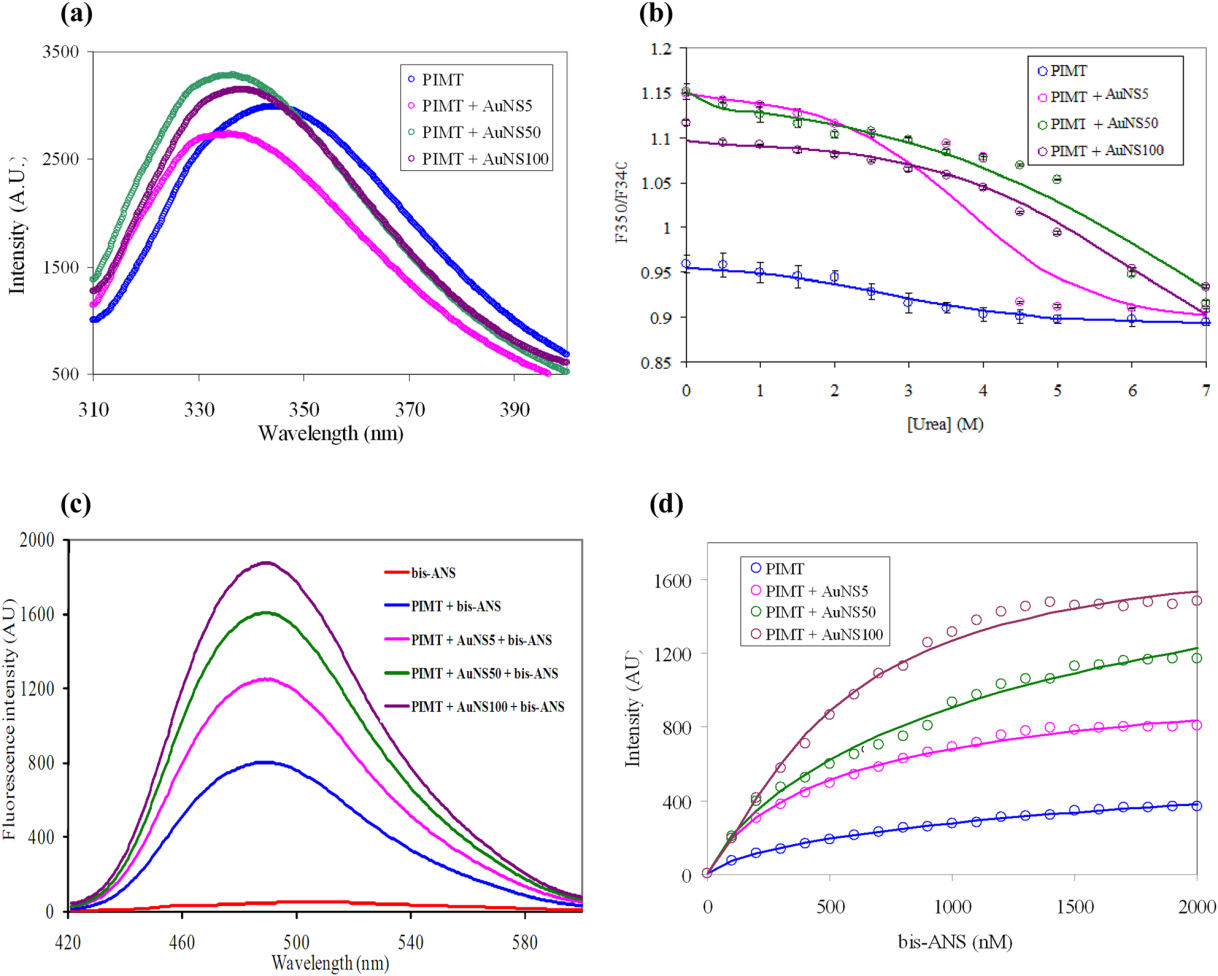
(**a**) Fluorescence spectra and (**b**) urea-induced unfolding of PIMT and PIMT in presence of AuNSs of different sizes in 1:1 molar ratio. Samples were excited as 295 nm in 0.1 M sodium phosphate buffer pH 7.2. (**c**) Fluorescence spectra of bis-ANS alone, and in presence of PIMT and PIMT-AuNS conjugates (in 1:1 molar ratio) in presence of bis-ANS. Protein concentration of 5 μM was used and the samples were excited at 395 nm. (**d**) Titration using fluorescence intensity against bis-ANS concentration, in presence of PIMT and PIMT-AuNS conjugates.

Recombinant PIMT has three tryptophan residues (Trp2, Trp222 and Trp225). The fluorescence emission wavelength maxima of recombinant PIMT appeared at 344 nm implying an exposure of the Trp residues to the polar environment. Interestingly, in presence of AuNSs the wavelength maxima exhibited blue shifts to 336, 337 and 339 nm respectively, for AuNS5, AuNS50 and AuNS100. The blue shifts in presence of AuNSs compared to PIMT may be indicative of the burial of tryptophan residues in the hydrophobic environment (Fig. 1a). The three Trp residues are close to the two termini of the polypeptide chain, which have been reported to have high mobility^21^, and are likely to be brought closer to the compact region of the molecule reducing their solvent exposure on AuNS binding.

Urea-induced unfolding was carried out by plotting the ratio of fluorescence intensities (*F*_*350*_*/F*_*340*_) against varying concentration of urea to compare the stability of PIMT in presence of AuNSs (Fig. 1b). PIMT got stabilized upon binding to all the three types of AuNSs as evident from the increase in the values of the free energy of unfolding *(ΔG*) and the midpoint of transition *[d]*_*1/2*_ (Table 1). However, AuNS100 was the most effective for PIMT stabilization as compared to its smaller counterparts, AuNS5 and AuNS50, implying the importance of the size of NP for modulation of protein stability. The efficacy of larger AuNSs was further validated from far-UV CD studies discussed below.

**Table 1:** Parameters obtained from urea induced unfolding of PIMT alone and in presence of AuNSs.

PIMT	Δ*G* (kcal mol^−1^)	[*d*]_*1/2*_ (M)	*m* (kcal mol^−1^ M^−1^)
Alone	1.6 ± 0.09	2.7	0.6 ± 0.34
+ AuNS5	2.5 ± 0.01	3.6	0.73 ± 0.02
+ AuNS50	2.0 ± 0.015	6.6	0.3 ± 0.03
+ AuNS100	2.7 ± 0.07	6.8	0.4 ± 0.02

### Effect of bis-ANS on PIMT and PIMT-AuNS conjugates

Tryptophan fluorescence spectra of PIMT exhibited blue shift of wavelength maxima upon treatment with all the three AuNSs implying exposure of tryptophan residues to a more hydrophobic environment (Fig. 1a). This prompted us to explore the effect of bis-ANS, an extrinsic fluorescent probe, whose intensity gets enhanced upon binding to hydrophobic residues in proteins. The bis-ANS fluorescence spectra of PIMT-AuNS conjugates showed enhanced fluorescence intensities as compared to PIMT only (Fig. 1c), indicating an inherent hydrophobic patch in PIMT that gets enlarged in presence of AuNSs.

To assess the binding affinity of bis-ANS to PIMT-AuNS conjugates titration was carried out by varying the concentration of the dye. Samples were excited at 395 nm and the fluorescence intensity at 495 nm, characteristics of bis-ANS was plotted showing exponential binding for PIMT alone, as well as for PIMT-AuNS conjugates (Fig. 1d). The binding affinity was calculated using Hill equation given below

1 $$ F_{{obs}}  = F_{{\max }} \left[ {\frac{{[bis - ANS]^{n} }}{{K^{\prime} + [bis - ANS]^{n} }}} \right] $$

where *F*_*obs*_ is the fluorescence intensity observed at a specific bis-ANS concentration, *F*_*max*_ is the maximum fluorescence intensity for PIMT as well as PIMT-AuNS conjugates, *K´* is the binding constant and *n* is the Hill coefficient, which also implies the binding stoichiometry. To evaluate *n* and *K’*, data were least-square fitted to Eq. (1) (supplementary information, Table S1). A value of ~ 1 is indicative of a single interaction site of bis-ANS for both PIMT, as well as PIMT-AuNS conjugates.

### Structural changes of PIMT upon binding to different AuNSs

**Figure 2:**
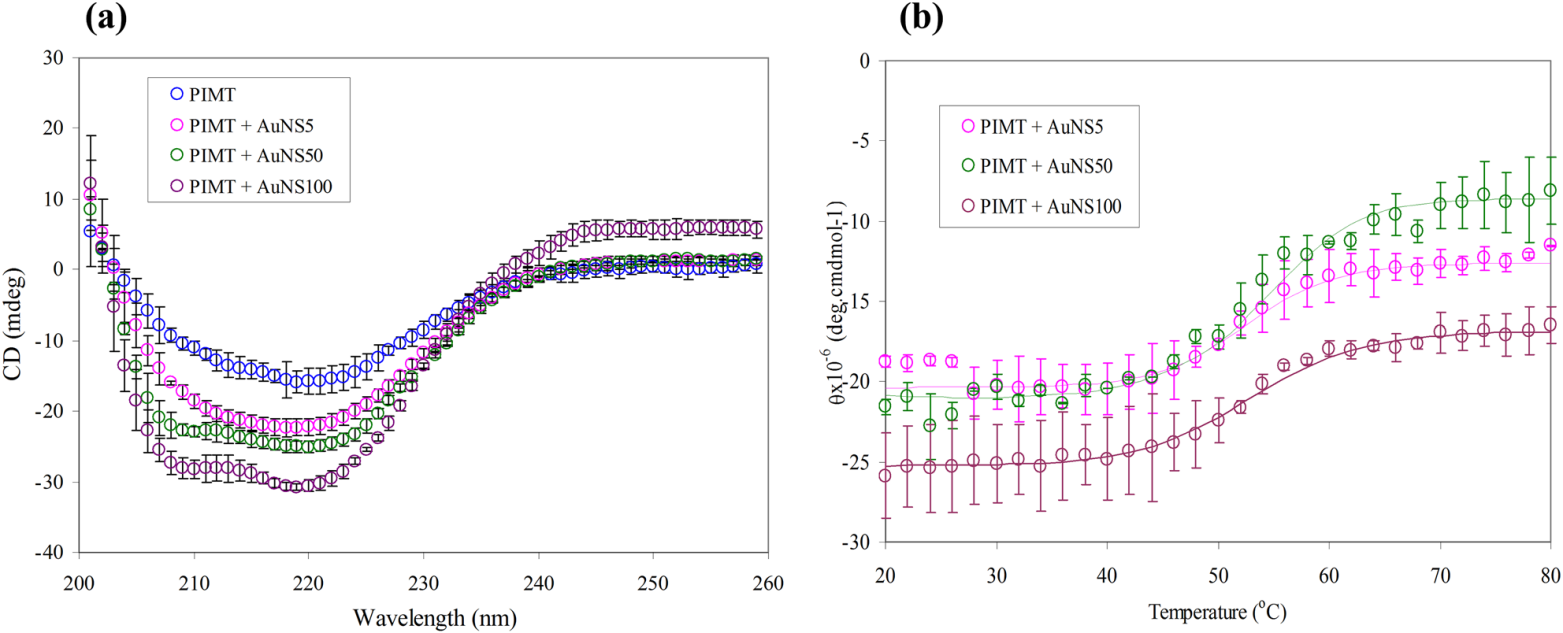
Far-UV CD of PIMT alone and in presence of (**a**) AuNSs of different sizes using 1:1 molar ratio in 0.1 M sodium phosphate buffer; pH 7.2 at 25 °C (**b**) Effect of temperature on the unfolding of PIMT in presence of AuNSs, as seen from the plot of the ellipticity at 222 nm.

Far-UV CD has been carried out to monitor the structural changes of PIMT induced by AuNSs of different sizes (Fig. 2a). The appearance of negative peaks at 208 and 222 nm are characteristics of α-helical structures, while the one at 218 nm indicates β-sheet structure. Deconvulation of CD spectra by CDNN revealed ~ 33% of α-helical and 35% random coil content of recombinant PIMT. Upon binding to AuNS5, AuNS50 and AuNS100 the α-helical content of PIMT got increased from 33 to 53%, 62% and 69%, respectively with the concomitant reduction of random coil indicating stability of PIMT (Table S2). This was further validated from temperature induced unfolding of PIMT and PIMT-AuNS conjugates by monitoring change in ellipticity with temperature (Fig. 2b)—the negative ellipticity values became less negative with the increase in temperature. The melting temperature (*T*_*M*_) was found to increase for all the three PIMT-AuNS conjugates as compared to the *apo* protein (52 °C), with the highest enhancement of 5 °C being in the presence of AuNS100. The results corroborate with the free energy of unfolding (*ΔG*) and the midpoint of transition *[d]*_*1/2*_ values (Table 1) obtained from the urea-induced unfolding of PIMT in presence of the AuNSs.

### Thermodynamics of binding of PIMT with AuNSs

**Figure 3:**
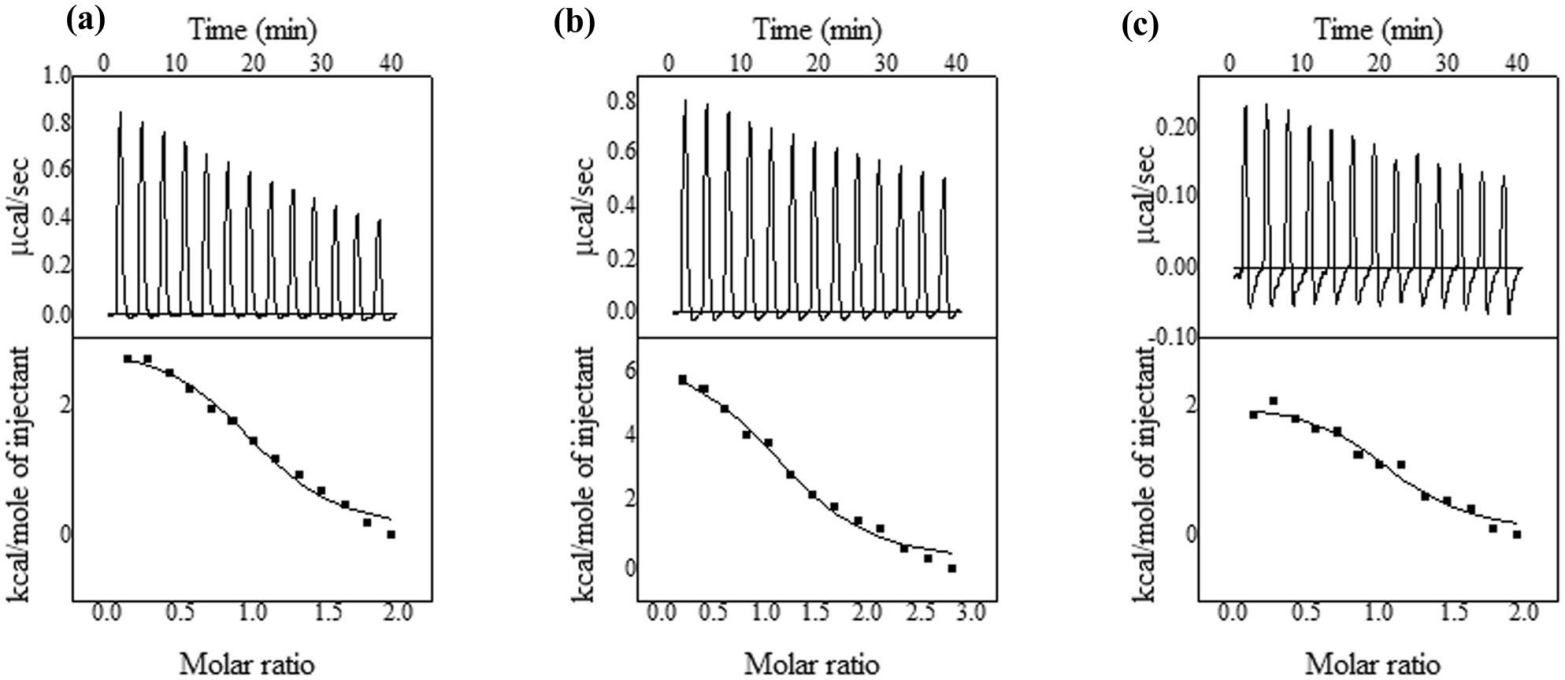
ITC data of titration of PIMT with (**a**) AuNS5, (**b**) AuNS50 and (**c**) AuNS100 using 20 mM of sodium phosphate buffer (pH 7.2) at 25 °C. The top panel shows the heat flow versus time for each injection of AuNSs while the bottom panel shows the heat evolved per mole of added ligands.

Isothermal titration calorimetry (ITC) is widely used for the evaluation of interactions between NPs and proteins^22^, and was employed here to probe the detailed thermodynamics and the mode of binding of AuNSs to PIMT (Fig. 3). The top panels show the heat signals generated from the interaction of injected AuNSs with PIMT in the sample cell; these were used to obtain a complete titration profile, given in the lower panels. The thermodynamics of reaction, along with the binding stoichiometry of protein-NP interactions are derived in Table 2. ITC revealed an endothermic mode of binding of PIMT with all the three spherical AuNSs. The unfavorable enthalpy change (*ΔH* > 0) may indicate the absence of any electrostatic or weak van der Waals interaction between PIMT and AuNSs. Favorable free energy of association between protein and NP has been known with endothermic heat change profile^23^. The positive values of entropy (*ΔS* > 0) imply a large hydrophobic component in the binding, presumably arising out of the release of a large amount of water of hydration from the binding interface, which finally leads to an overall favorable change of Gibbs free energy (*ΔG* < 0). Thus the binding is entropy controlled. The binding constant *K* is about 2–4 times higher with AuNS100 relative to the smaller counterparts (AuNS5 and AuNS50). This observation is in accordance with the literature where stronger binding of proteins was achieved with larger NPs^24^.

**Table 2:** Thermodynamics of binding of PIMT to AuNSs of different sizes.

Parameters	AuNS5	AuNS50	AuNS100
*N* (stoichiometry)	1.04 (± 0.04)	1.23 (± 0.05)	1.1 (± 0.05)
*K* (binding constant, M^−1^)	3.96 (± 1.03) × 10^5^	7.34 (± 1.8) × 10^5^	1.23 × 10^6^ (± 4.7 × 10^5^)
Δ*H* (binding enthalpy, kcal/mol)	2.97 (± 0.15)	6.73 (± 6.6)	2.07 (± 0.13)
*ΔS* (entropy change, cal/mol.K)	35.4	49.0	54.7
Δ*G* (free energy change, kcal/mol)	− 7.6	− 7.87	− 14.23

### Binding of PIMT to AuNSs using dynamic light scattering (DLS)

The strong light-scattering intensity of AuNPs enables DLS to be used as a sensitive tool to monitor adsorption of protein to AuNPs. We used DLS to probe the change in diameter of PIMT after conjugation with AuNSs (Fig. S3 and Table S3). PIMT has a diameter of 4.9 nm, which got increased upon conjugation with all AuNSs. For the PIMT-AuNS5 conjugate the diameter was found to be 10.9 nm, while that for PIMT-AuNS50 it was 61.03 nm. The largest AuNS100 showed a value of 121.2 nm after binding to PIMT. The diameters were used to calculate the average number of PIMT molecules that can fit on to AuNSs of different sizes^25^. The maximum number of PIMT that can bind to AuNSs was calculated by dividing the surface area (surface area = $\pi d^{2}$) of the difference of PIMT-AuNS conjugates from that of the respective AuNSs by the surface area of the protein (Table S3). This gives the possible number of bound PIMT molecules, 4 for AuNS5, 82 for AuNS50 and 230 for AuNS100.

### Enhancement of methyltransferase activity of PIMT in presence of AuNSs

The methyltransferase activity *(M)* of the recombinant PIMT on Aβ42 as substrate was calculated using the following equation

2 $$ M = \frac{{absorbance/\min }}{{15.0}} \times \frac{{0.1}}{{0.01}} $$

The activity was calculated from the rate of change of formation of 3,5-dicholoro-2-hydroxy-benzenesulfonic acid (DHBS) at 510 nm. All the spherical AuNPs enhanced the activity (Fig. S4). On its own PIMT showed an activity of 9000 pmol/min/mg, which got enhanced to 16,000 pmol/min/mg and 18,000 pmol/min/mg, respectively, when used with AuNS5 and AuNS50. Along with AuNS100, PIMT exhibited the highest activity of 24,000 pmol/min/mg, showing once again the efficacy of the largest AuNS.

### ThT assay for Aβ fibrillation using PIMT and PIMT in presence of AuNSs

**Figure 4:**
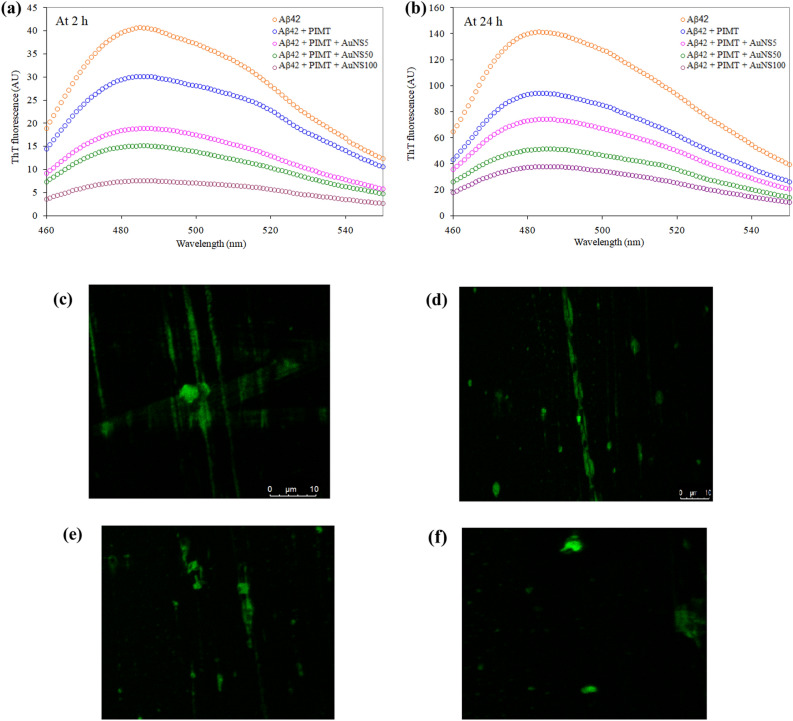
Thioflavin T fluorescence spectra of Aβ42 alone, and after treatment with PIMT and PIMT in presence of AuNSs after (**a**) 2 h and (**b**) 24 h. Samples were excited at 440 nm in 20 mM sodium phosphate buffer (pH 7.2). (**c**)–(**f**) Confocal microscopic images of Aβ42 fibrils under different conditions, (**c**) peptide alone, and in presence of (**d**) PIMT, (**e**) AuNS100 and (**f**) PIMT and AuNS100 together.

To substantiate the effectiveness of PIMT-AuNS conjugate for reduction of fibrillation, we used ThT fluorescence assay to study the fibrillation of Aβ42. The dye ThT which is non-fluorescent in buffer solution becomes highly fluorescent on binding to fibrils, when excited at 440 nm^26^. For Aβ42, the enhancement of ThT fluorescence was observed with the increase in incubation time (2 h and 24 h) (Fig. 4a,b) implying aggregation of the peptide. The extent of fibrillation of Aβ42 was inhibited after treatment with PIMT, as evident from the reduction of fluorescence intensity at 2 h that continued till 24 h, implying reduction of fibrillation. This effect was further enhanced when PIMT was added along with AuNSs of different sizes, showing the synergistic effect of PIMT and AuNSs for inhibition of fibrillation. It may be added that AuNSs by themselves do not have much effect on the fibrillation features of Aβ42 (Fig. S5).

### Reduction of fibril formation of Aβ42 by PIMT and PIMT in presence of AuNS100

Since AuNS100 was found to be the most effective for the stabilization of the structure of PIMT, the remaining experiments were carried out using AuNS100 only. Representative images of fibrils formed by Aβ42 and the effect of PIMT, AuNS100 and PIMT and AuNS100 together, as observed from confocal microscopy are shown in Fig. 4c–f. Reduction of fibril formation can be seen using both PIMT and AuNS100 by themselves, which was further enhanced with PIMT-AuNS100 conjugate.

### On the binding site of AuNS on PIMT and the effect on the enzyme structure

**Figure 5:**
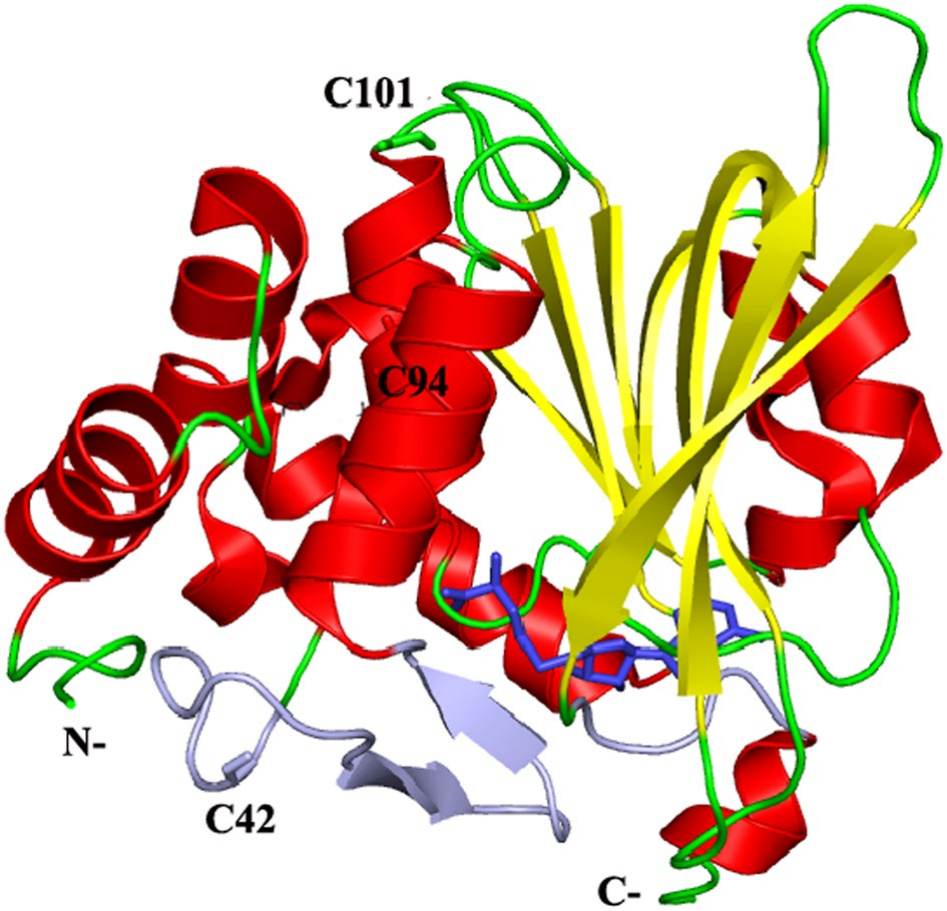
Cartoon representation of human PIMT structure, with the secondary structures, helix (red), sheet (yellow) and loop (green), shown in different colors, and the cofactor (S-adenosyl homocysteine) shown in stick model, based on the PDB file, 1I1N). The N- and C-termini of the polypeptide chain, as well as the three Cys residues in the protein are labelled. The regions lining the binding site, and towards the exterior of the molecule, are indicated in light violet—these include residues Thr57-Ser59 and Tyr212-Thr216. (As in some structures the residues upstream of the former region are found to be rather flexible or disordered, the entire stretch, Lys41-Ser59 has been colored).

Human PIMT is a monomeric protein, 226 residue long (PDB ID: 1I1N)^21^ with isoelectric point (pI) of 6.42 as calculated from ProtParam tool (www.expasy.org/cgi-bin/protparam/). AuNSs used in the present study have pI of 7.0 which ruled out any type of protein–ligand electrostatic interaction. PIMT contains three cysteine residues (Cys42, Cys94 and Cys101) and these residues may bind to AuNS via the formation of a coordinate covalent bond between sulfur and gold, as Au–S bond formation is energetically favorable^27,28^. Among different PIMT sequences Cys42 and Cys101 are not conserved, Cys94 is^21^. Cys94 is buried in the structure (with relative solvent accessibility, 2.5), but the other two are exposed (Cys42, 64.6; Cys101, 79.2). While Cys101 is quite away, Cys42 is close to the putative binding site of AuNS (Fig. 5). However, a positive *ΔH* (Table 2) may possibly suggest that the Cys residue is not involved in coordinating Au.

Antiepileptic drugs have been shown to exert allosteric effect on the activity of PIMT, binding close to the active site^10^. It is also possible that AuNS binds to a region around the active site. In human PIMT a cleft is formed between the N-terminal domain and the C-terminal end of the central sheet domain^21^. The putative isoAsp substrate binding site has been proposed to be formed by the sides of the cleft (in particular conserved residues 57–59 and 212–216). However, to bind diverse isoAsp-containing proteins other nearby regions in PIMT are also likely to be used, and in many proteins, such as *Vibrio cholera* PIMT (which has activity similar to mammalian PIMTs) residues 39–58 are disordered in the *apo* state^7^. The putative substrate binding site is thus at the bottom of the displayed molecule (Fig. 5). It is likely that AuNSs binding contributes to the ordering near the substrate binding region, thereby increasing the activity. The N- and C-termini regions, which in general are very flexible, are close by and are also likely to get more ordered. It has been shown that when a protein binds to its cognate partner (another protein or DNA), there may be disorder to order transition, or irregular regions taking up secondary structures, and the most observed increase in secondary structure is the extension of already existing helices (or strands)^29,30^. It is pertinent to note that the binding of AuNSs has also been observed to increase the helical content of PIMT (Table S2).

### Cytotoxicity of PIMT and AuNSs in PC12 derived neurons

We initially checked the cytotoxic effects of PIMT and the various AuNSs on the PC12 derived neurons before performing the cell rescue experiment with Aβ42. For this, PC12 cells were plated in 96 well plates and differentiated into neurons using NGF medium for another 5 days. After the neuronal structures were observed in the plates, the cells were treated in varying concentrations (3.12, 6.25, 12.5, 25, 50 and 100 μM) of PIMT and (3.12, 6.25, 12.5, 25, 50 and 100 μM) of AuNSs for 24 h. Thereafter, the cytotoxicity index of the treated compounds was analyzed by performing the standard MTT assay and it was clearly evident from the results (Fig. S6) that neither the enzyme nor the AuNSs exhibited any toxic effects on the neurons.

### Cell rescue assay in PC12 derived neurons

**Figure 6:**
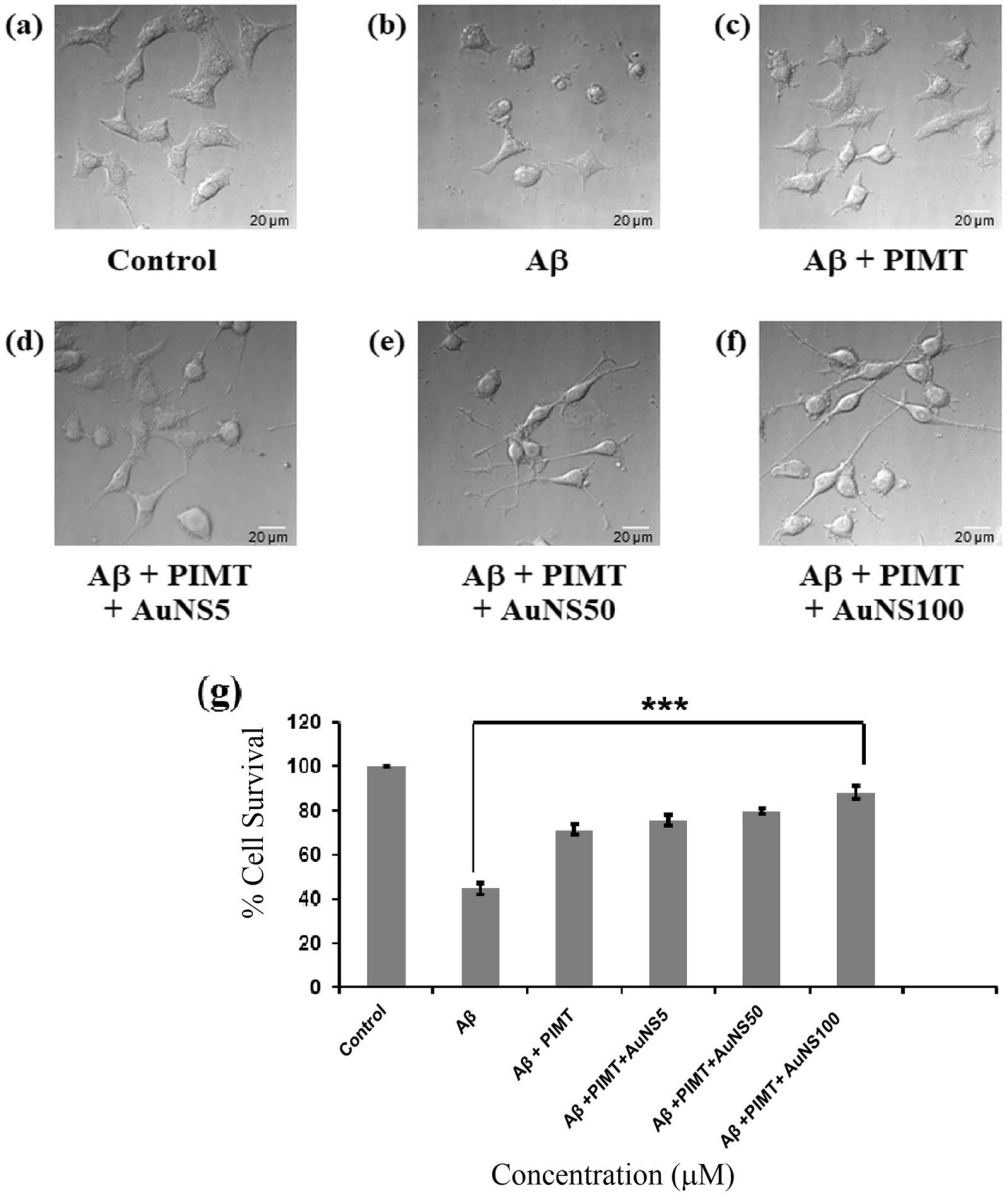
Microscopic images of (**a**) untreated control, (**b**) Aβ42 treated, Aβ42 treated along with (**c**) PIMT, and PIMT in presence of (**d**) AuNS5, (**e**) AuNS50 and (**f**) AuNS100, taken in DIC mode (scale bar corresponds to 20 μm). (**g**) Data, showing the percentage survival for the Aβ42 study with PIMT and PIMT-AuNS conjugates in PC12 cell derived neurons. The results have been statistically analyzed by 1-way ANOVA (***P < 0.0001, n = 4).

So far we have reported the anti-amyloidogenic nature of PIMT and PIMT-AuNS conjugates using various in vitro studies. Next we validate these findings in an Aβ42 infused cellular model. In this experiment, we plated PC12 cells again in a 96 well plate and differentiated it with nerve growth factor to produce neuronal architecture. The neurons are then treated with 10 μM Aβ42 alone, as well as with PIMT, and PIMT-AuNS conjugates, for 24 h. The cell survivability was 45% in presence of Aβ42 (Fig. 6g). PIMT alone reduced the amyloid toxicity and promoted neuronal survival by showing 60% recovery compared to the value for the Aβ42 affected neurons (Fig. 6g). The recovery percentages were even higher, 69%, 78% and almost 97%, when treated with PIMT-AuNS conjugates involving AuNS5, AuNS50 and AuNS100, respectively (Fig. 6g). The microscopic images (Fig. 6a–f) also reflected the same—as compared to the healthy neuronal morphology in the control, untreated cells (Fig. 6a), we saw crippled neuronal morphology in 10 μM Aβ42 treated neurons (Fig. 6b), which got substantially improved in all the PIMT treated cells, showing more healthy neuronal morphology (Fig. 6c). With the PIMT-AuNS conjugates the neuronal morphology gradually improved with the increasing size of AuNS—it exhibited good morphology in PIMT-AuNS5 conjugate (Fig. 6d), which got better in PIMT-AuNS50 (Fig. 6e). But the best morphology resulted from the treatment with PIMT-AuNS100 conjugate, where long neuronal branching was observed, leading to synaptic interconnections and neuronal network formation (Fig. 6f). These results in the neurons are a testament to our earlier observations and demonstrate that PIMT-AuNS100 conjugate can serve as an excellent anti-amyloidogenic agent^31–35^.

## Discussion

Aβ42 peptide has been implicated in fibril formation that is the leading cause of many neurodegenerative diseases. We had shown that the enzyme PIMT can reduce the fibril formation in an isoAsp-containing peptide and this can be of general significance in all biologically relevant proteins that contain natural Asp or Asn residues that can get converted to isoAsp on ageing^10^, Aβ42 being one such molecule. In this work we studied if three AuNPs of different sizes (5, 50 and 100 nm) can have an augmenting effect on the structure and antifibrillation activity of PIMT. Far-UV CD spectra showed that there was an increase in α-helical content at the expense of random coil in presence of all the AuNSs (Fig. 2, Table S2), AuNS100 exhibiting the maximum efficacy in stabilizing PIMT. The *T*_*M*_ values also showed an increase for PIMT-AuNS conjugates compared to the *apo* protein (Table S2). Even chemical unfolding of PIMT in presence of all three AuNSs revealed stabilization of the structure, irrespective of the size of NPs as evident from the free energy of unfolding (*ΔG*) as well as from the midpoint of transition *[d]*_*1/2*_ values (Table 1). Interestingly, the activity assay (Fig. S4) indicated that commensurate with the changes in the structure, the activity of PIMT increased in presence of AuNPs, AuNS100 showing the maximum enhancement. In addition we have used DLS to monitor the conjugation of PIMT on AuNSs. There are reports in literature indicating that for proteins having at least 3 nm diameter, the conjugation on AuNPs can easily be detected by DLS^36^. In our study, PIMT, having a hydrodynamic diameter of 4.9 nm, showed an increase in the magnitude upon conjugation with all the AuNSs. This also enabled us to calculate the approximate number of PIMT molecules bound to different AuNSs (Table S3).

The extent by which a protein undergoes NP-induced conformational change defines it as a “hard” or “soft” protein^37,38^. Proteins whose structures got compromised after treatment with NPs are designated as “soft” while those retaining the functional native conformations are known as “hard”. In the present study PIMT has been found to retain its native-like conformation and activity, though some of the irregular regions in the molecule are likely to have been transformed to helical structure, with all the three spherical AuNSs. This may represent a case that is somewhat in between these two extreme situations.

The size of nanoparticle is a crucial parameter affecting the structure and function of adsorbed proteins^39,40^. Larger AuNPs having low surface curvature provide lower surface area of interaction compared to those of smaller size with high surface curvature. Adsorption of PIMT was found to be more with the smallest AuNS5 compared to the larger AuNS50 and AuNS100 (Fig. S2), as was also reported for the surface coverage of dihydrofolate reductase on AuNPs with diameters of 5, 15 and 30 nm^25^. Previous studies using various model proteins, such as lysozyme, human serum albumin, ribonuclease A reported retention of native-like structure and hence function in presence of smaller nanoparticles^41–43^. However, it is difficult to generalize as protein-NP interaction and the consequent conformational changes depend on the intrinsic properties of that particular protein^24^. Graphene oxide was found to have different effect on thermal (and storage) stability of a number of proteins, and with lysozyme, though there was no significant change in structure, the binding near the active site affected the flexibility of the surrounding residues and contributed to the reduction of the activity^44^. In the present study we find that among the three AuNSs, the largest, AuNS100 showed the maximum stabilization of the structure and anti-fibrillation activity of PIMT, whereas we had earlier reported that the same AuNS100 compromised the structure/function of a toxic protein (less than half the size of PIMT), Accessory cholera enterotoxin (Ace) of *Vibrio cholera*, but the smaller AuNS10 did not have much influence on the structure^45^.

There are flexible regions in PIMT, especially at the C-termini of the chain, near the substrate binding site (Fig. 5). It is possible that AuNS binds near the active site, bringing in ordering of the flexible regions, as was inferred from other biophysical studies. There is negative change in Gibbs free energy and the binding is entropy controlled (Table 2). Consistent with the other results AuNS100 has a higher binding constant compared to the two other smaller AuNSs. Using ThT fluorescence study it was found that the fibrillation of Aβ42 was reduced, progressively to a greater extent when PIMT was used in conjunction with AuNSs of higher size (Fig. 4). The anti-amyloidogenic nature of PIMT-AuNS conjugates was then validated in a cellular model. The neuronal architecture was disturbed in PC12 derived cells infused with Aβ42. This could be recovered to a considerable extent on treatment with PIMT and more so with PIMT-AuNS conjugates, the revival matching approximately with the size of AuNSs, the maximum restoration achieved with AuNS100 (Fig. 6).

Interaction of NPs with proteins is known to induce cooperative effects, such as promotion or inhibition of protein fibrillation or self-assembling of NPs on macromolecules serving as a template^39^. Our study brings out an indirect effect of NP on protein fibrillation by bringing out changes in the structure/activity of an enzyme influencing its role in preventing fibrillation. That AuNPs can enhance the specific activity of an enzyme, such as α-amylase in a concentration-dependent manner has also been reported^46^.

## Conclusions

The in-depth knowledge of protein-NP interaction and how the NP exerts its influence in changing the structure and function of the protein can usher in fascinating roles for nano-bioconjugates in biomedical applications^47,48^. Here we report the effect of different size of spherical AuNPs viz*.* AuNS5, AuNS50 and AuNS100 on the structure and anti-fibrillation activity of PIMT. Brain proteins with Asp and Asn are endogenous substrates for PIMT^49^. We have recently shown that isomerizing these altered residues back to the native state by PIMT can prevent protein fibrillation, showing how an enzyme can have an important role in ameliorating the root cause of neurodegenerative diseases^10^. In this paper we further confirm the beneficial role of PIMT and how it can also be boosted when used as PIMT-AuNS conjugates. When PIMT-AuNS100 conjugate was administered to Aβ42 induced PC12 derived neurons, it was seen that as compared to PIMT alone, PIMT-AuNS100 conjugate promoted the cellular survival of the derived neurons to a much greater extent, thereby clearly demonstrating the in vivo potential of this combination in the betterment of Aβ42 induced cellular toxicity.

## Materials and methods

### Materials

Gold nanospheres 5 nm (product code # 752568), 50 nm (# 753645) and 100 nm (# 753688) as well as Aβ42 peptide were purchased from Sigma Aldrich, USA. The AuNSs were stored at 4 °C and used as supplied. Ni–NTA agarose was obtained from Qiagen. IPTG and PMSF were purchased from Sigma Aldrich, USA. Urea was purchased from Calbiochem, India. All other chemicals were of analytical grade and were procured from Merck, India. Horse Serum was bought from Himedia Laboratories. Fetal Bovine Serum (FBS), trypsin–EDTA, penicillin and sterptomycin were obtained from Gibco (Thermo Fisher Scientific). Thiazolyl Blue, tetrazolium bromide (MTT), DMEM low glucose medium and Nerve Growth Factor-7S were purchased from Sigma Aldrich.

### Purification of recombinant PIMT

Recombinant PIMT was purified as described earlier using Ni–NTA affinity chromatography and visualized by running into 10% Tris-tricine gel followed by coomassie blue staining^10^. The fractions containing pure protein was pooled and dialyzed against 0.1 M sodium phosphate buffer pH 7.2. Concentration of protein was estimated spectrophotometrically using a molar extinction coefficient of 27,055 M^−1^ cm^−1^ at 280 nm.

### SPR spectroscopy for PIMT-AuNS interaction

SPR spectra of AuNSs of different sizes and PIMT-AuNS conjugates were recorded in Hitachi U-2900 spectrophotometer using a quartz cuvette of 1 cm pathlength. The spectra were recorded in the visible range from 400 to 800 nm for all AuNSs and their conjugates with PIMT.

### Adsorption of PIMT to AuNSs by UV–Vis spectroscopy

Adsorption of PIMT was measured using different concentrations of protein (2, 4, 6, 8, 10, 25, 35, 45 and 50 µM) in 0.1 M sodium phosphate buffer (pH 7.2). All samples were incubated at 25 °C with 50 µM of AuNSs for 30 min. Samples were then centrifuged at 13,000 rpm for 15 min. Control sets without AuNSs were prepared with only PIMT using requisite concentrations as mentioned above. The supernatants were taken in a quartz cuvette of 1 cm pathlength and absorbance was measured in Hitachi U-2900 spectrophotometer at 280 nm. The difference in absorbance (*ΔA*) at a specific concentration of PIMT is given as

$$ \Delta A = A_{{{\text{control}}}}  - A_{{{\text{supernantant}}}} $$

### Fluorescence spectroscopy

Fluorescence measurements were carried out in Hitachi 3000 spectrofluorimeter. For tryptophan fluorescence protein concentration of 5 µM was used in 0.1 M sodium phosphate buffer (pH 7.2). Samples were excited at 295 nm and emission was measured in the range of 310–420 nm using slit widths of 5 nm. Urea induced unfolding of PIMT and PIMT in presence of AuNSs of different size was carried out by incubating with varying concentration of urea at 25 °C. Analysis of data was carried out by assuming two-state model of folded to unfolded [Folded (F) → Unfolded (U)] state of protein using the following equation

$$ \Delta G = \Delta G^{{H_{2} O}}  - m[d]_{{1/2}} $$

where *ΔG* is the free energy of unfolding, *ΔG*^*H*^_*2*_^*O*^ is the change in free energy in the absence of urea, *[d]*_½_ is the mid-point of transition which is obtained by dividing *ΔG* by the slope.

bis-ANS is an external fluorescent probe which is frequently used to monitor exposed hydrophobic residues^50^. bis-ANS titrations of PIMT in presence of spherical AuNSs (using 1:1 molar ratio) were conducted by exciting the samples at 395 nm and the emission was monitored in the range of 420–600 nm. Protein concentration of 5 µM was used.

### Circular dichroism spectropolarimetry

CD spectra of PIMT and PIMT with AuNSs (using 1:1 molar ratio) were carried out in a JASCO-810 spectropolarimeter equipped with a thermostated cell holder. Scans were taken in the wavelength range of 200 to 260 nm in a quartz cuvette of 1 mm path-length with a scan speed of 100 nm/min. For each sample five individual scans were acquired and the spectra were averaged. Deconvolution of CD spectra was carried out using CDNN software (http://thelab.photophysics.com/circular-dichroism/protein-secondary-structure-analysitools-cdnn/) ^51^.

Thermal unfolding of samples was recorded in the range of 20 to 80 °C in steps of 2 °C. The melting temperature (*T*_*M*_) was calculated by plotting mean residue ellipticity (MRE) against temperature. The observed ellipticity at 222 nm (*θ*_222_) was converted to MRE using the following equation

$$ [\theta _{{222}} ] = 100\theta Mw/cln $$

where *Mw* is the molecular weight of PIMT, *c* is the concentration of protein in mg/ml, *l* is the path length of the cuvette in cm and *n* is the number of amino acids. For the calculation of *T*_*M*_ the Gibbs–Helmholtz equation was used^52^

$$ {\Delta G = \Delta H\left( {{\text{1}} - T/T_{M} } \right) - \Delta C_{p} T_{M} \left[ {{\text{1}} - \left( {T/T_{M} } \right) + \left( {T/T_{M} } \right){\text{ln}}\left( {T/T_{M} } \right)} \right]} $$

where *T*_M_ is the melting temperature, Δ*H* is the change in enthalpy, and Δ*Cp* is the change is specific heat capacity from the folded to the unfolded state.

### Binding of AuNSs to PIMT using isothermal titration calorimetry (ITC)

ITC was carried out in a VP-ITC calorimeter (Microcal Inc., Northampton MA). PIMT was thoroughly dialyzed in 0.1 M KP (pH 7.2) and the AuNSs were diluted using the last dialysate. Titration consisted of 13 injections of AuNSs as ligands (injection volume 10 μl, conc. 100 µM) into the sample cell containing PIMT (volume 1.4 mL, conc. 10 µM) and stirred continuously at 310 rpm. The data were analyzed using the Origin 7.0 software to obtain the binding stoichiometry (*n*), binding constant (*K*), enthalpy (*ΔH*) and entropy (*ΔS*) of reactions.

### Dynamic light scattering (DLS)

Dynamic Light Scattering (DLS) was used for determination of change in hydrodynamic radius of PIMT after conjugation with AuNSs. DLS was carried out using Zetasizer Nano Malvern Instrument (UK) using a laser source of 633 nm. Three DLS measurements were conducted for each sample with 15 runs and the duration for each run was 15 s. 0.1 M sodium phosphate buffer (pH 7.2) was used and both buffer and the protein sample was filtered through 0.22 micron filter unit prior to use.

### Preparation of Aβ42 fibril for visualization using confocal microscopy

The stock of lyophilized Aβ42 was dissolved in hexafluoro-2-pronanol (HFIP) and distributed into small aliquots. After removal of HFIP, the aliquots were stored at − 80 °C till further use. The lyophilized peptide sample was dissolved in 2 mM NaOH to make a stock of 1 mg/mL prior to experiment. For confocal microscopy, 30 µM of Aβ42 alone and in presence of PIMT, AuNS100 and PIMT with AuNS100 (using a molar ratio of 1:1) were incubated at 37 °C for 24 h. 10 µl of each sample was placed on clean glass slides followed by addition of thioflavin T (ThT) dye and allowed to dry in dark under laminar flow. Samples were sealed with cover slips using DPX mounting media and visualized in a confocal microscope (Model: Leica TCS SP8) using a 405 nm band pass filter.

### Thioflavin T spectroscopy

Stock solution of ThT was prepared by dissolving in Milli Q water, the concentration of which was measured from UV absorbance at 412 nm using a molar extinction coefficient of 36,000 M^−1^ cm^−1^. ThT fluorescence was measured in JASCO FP8500 spectrofluorimeter at an excitation/emission of 440/485 nm, respectively. Samples of Aβ42 were incubated for different time intervals at 37 °C and ThT fluorescence intensities were measured in presence of PIMT and PIMT conjugated with different AuNSs (using 1:1 molar ratio).

### Methyltransferase activity assay of PIMT in presence of AuNSs

The impact of AuNSs on the methyltransferase activity of PIMT was assayed using SAM-510 colorimetric assay kit (G-Bioscience, https://www.gbiosciences.com/) as described earlier^10^. Briefly the enzyme was incubated with Aβ42 peptide as substrate alone or in the presence of AuNS5, AuNS50 and AuNS100 using 1:1 molar ratio in Tris–HCl buffer (0.1 M, pH 7.2). A “master mix” was prepared following manufacturer’s protocol and reaction was initiated by adding the “master mix” to protein-peptide mix. The absorbance at 510 nm was measured at different time intervals using a Fluostar Optima microplate reader.

### Culturing of PC12 cells

PC12 cells are basically rat adrenal pheochromocytoma cells which we cultured in DMEM medium (low glucose) and to which 10% horse serum, 5% fetal bovine serum and 1% penicillin–streptomycin solution were added. Then the cells were kept overnight in an incubator at 37 °C in an atmosphere containing 5% CO_2_. The cells were further differentiated into neurons in a special neuron inducing medium, consisting of low glucose DMEM, 1% horse serum and 100 ng/mL NGF (nerve growth factor), for around five days. All the assays have been performed using these neurons obtained from PC12 cells.

### Cellular toxicity assay

To assess the toxicity of the PIMT and AuNSs, MTT [3-(4,5-dimethylthiazol-2-yl)-2,5-diphenyltetrazolium bromide] assay was carried out in cellular models. In this assay, the tetrazolium dye MTT forms an insoluble formazan as an indicator for the percentage of live cells by using cellular reductases. For this assay PC12 cells were plated in 96 well plates, differentiated to produce neurons and then treated with AuNSs. After 24 h, MTT dye was added to each well leaving a few untreated wells for auto fluorescence correction. The insoluble formazan produced after 4 h was dissolved in a mixture of DMSO: methanol in 1:1 ratio and the absorbance was measured at 570 nm using a plate reader and the percentage of cellular viability was plotted.

### Cell survival assay with Aβ42 in PC12 derived neurons

The differentiated PC12 cells in 96 well plates were treated with 10 μM Aβ42 along with 1 μM of PIMT and PIMT-AuNS conjugates for 24 h. Cell survival percentage was measured after 24 h using the aforementioned MTT assay. The neuronal morphology of the treated and control neurons were captured in DIC mode at 40 X magnification using Olympus (IX83) microscope provided with an Andor iXon3 897 EMCCD camera.

## Supplementary Information

Supplementary Information.
