# Supplementary material for: Effect of gold nanoparticles on the structure and neuroprotective function of protein L-isoaspartyl methyltransferase (PIMT)
Source: Sci Rep. 2021 Jul 12;11:14296. doi: 10.1038/s41598-021-93752-1 (PMC8275801; doi:10.1038/s41598-021-93752-1)
Supplement: Supplementary file 1 — Supplementary Information. [file 41598_2021_93752_MOESM1_ESM.pdf]

Effect of Gold Nanoparticles on the Structure and Neuroprotective Function of Protein *L*-isoaspartyl methyltransferase (PIMT)

Tanaya Chatterjee, Gaurav Das, Surajit Ghosh and Pinak Chakrabarti

Supplementary information

Table S1. Binding of bis-ANS to PIMT and PIMT-AuNS conjugates

	PIMT			
	**Alone**	**+ AuNS5**	**+ AuNS50**	**+ AuNS100**
Hill coefficient (*n*)	0.72	0.85	0.82	1.3
Binding constant (*K′*, μM)	0.33	0.26	0.44	3.5

Table S2. Secondary structure contents[a] and melting temperatures of PIMT alone and PIMT in presence of AuNSs of different sizes

	**Alone**	**+ AuNS5**	**+ AuNS50**	**+ AuNS100**
θ_{222}	-15.6 ± 1.9	-21.7 ± 1.2	–24.5 ± 1.0	-29.7 ± 1.07
α-helix (%)	32.6 ± 1.9	52.5 ± 3.4	62.3 ± 2.1	68.7 ± 3.4
Random coil (%)	35.1 ± 2.1	23.3 ± 1.5	17.7 ± 1.01	14.5 ± 1.2
Melting temp. (T_M, °C)	52.0[b]	53.0	54.0	57.0

[a] Based on the deconvulated CD data.

[b] From ref # 10

Table S3. Diameter of PIMT, AuNSs and PIMT-AuNS conjugates, obtained from DLS measurements (Fig. S3), and the number of PIMT molecules bound to AuNSs

Sample	Diameter (nm)	Surface area (nm^2)	Number of PIMT bound
AuNS5	5.9	109	-
AuNS50	41.8	5,490	-
AuNS100	95.7	28,776	-
PIMT	4.9	75	-
PIMT + AuNS5	10.9	373	4
PIMT + AuNS50	61.03	11,703	82
PIMT + AuNS100	121.2	46,154	230

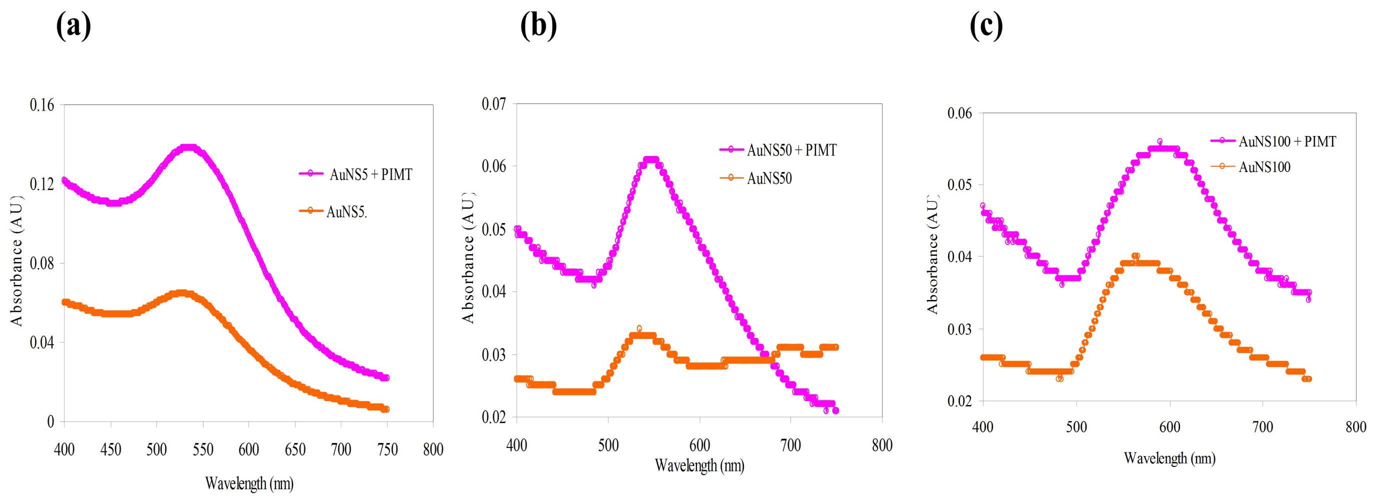

Fig. S1. Surface Plasmon Resonance (SPR) absorption bands of AuNSs of different sizes and the changes brought about by binding to PIMT.

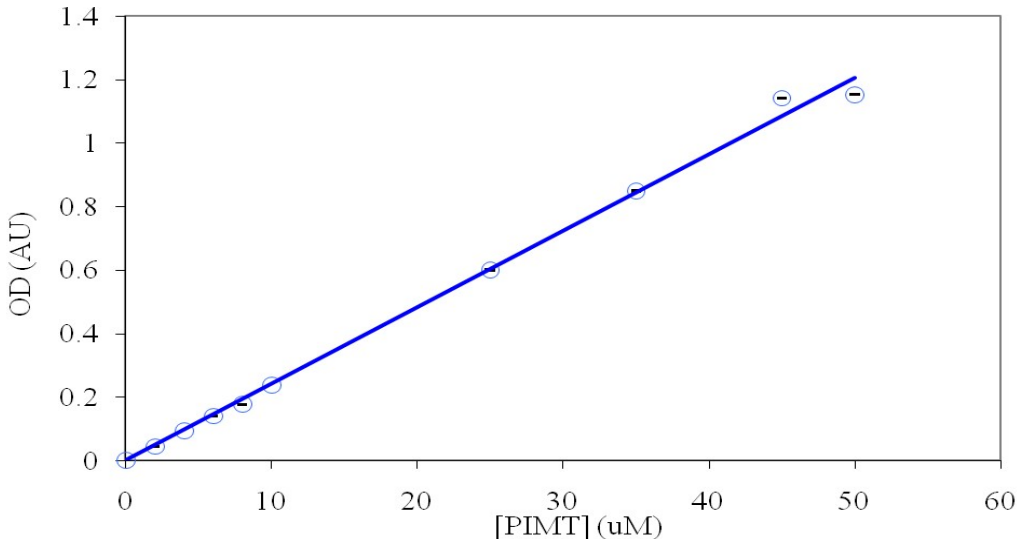

(a)

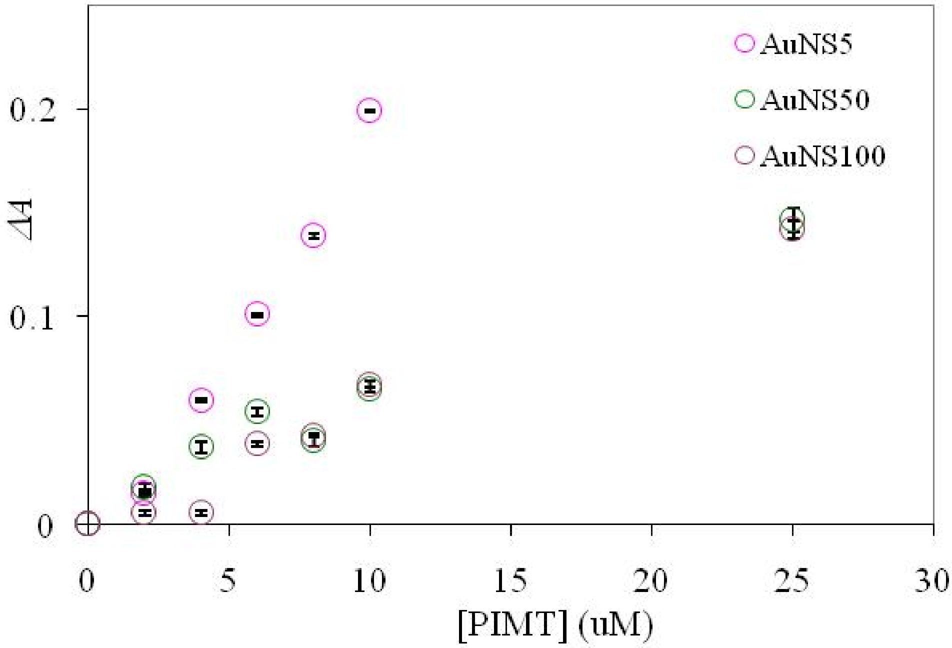

(b)

Fig. S2. OD values of PIMT on AuNSs of different sizes. (a) OD_{280} of PIMT alone with increasing concentration. (b) Difference in OD values of PIMT-AuNSs conjugates from free PIMT.

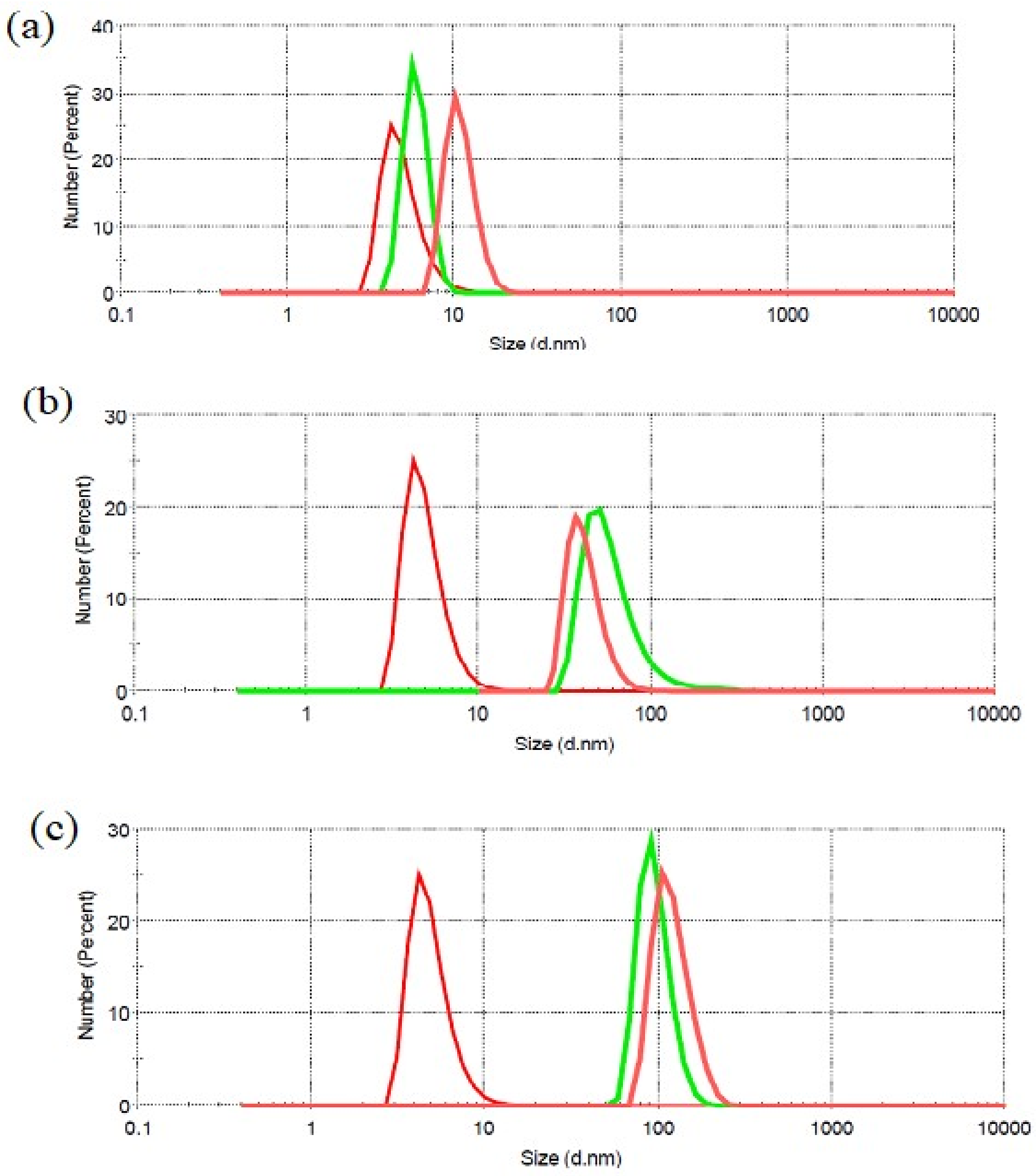

Fig. S3. DLS of AuNSs and PIMT individually, and their conjugates. (a) AuNS5 (green) and PIMT-AuNS5 (pink), (b) AuNS50 (pink) and PIMT-AuNS50 (green) and (c) AuNS100 (green) and PIMT-AuNS100 (pink). PIMT alone in all is shown in red.

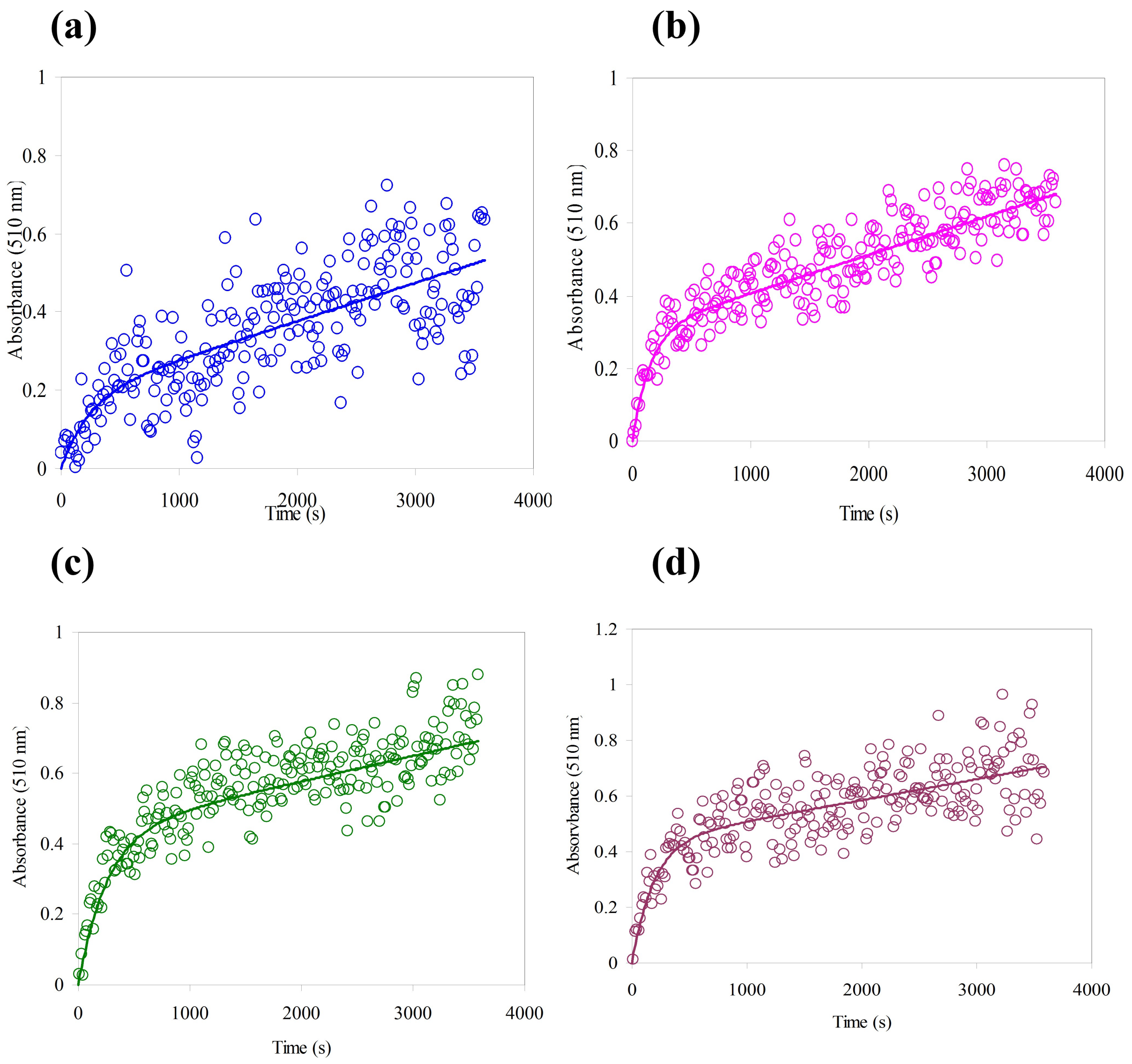

Fig. S4. *In vitro* methyltransferase activity assay of PIMT (a) alone, and in presence of (b) AuNS5, (c) AuNS50 and (d) AuNS100, on Aβ42 substrate, using SAM methyltransferase assay kit. A representative of three measurements is shown.

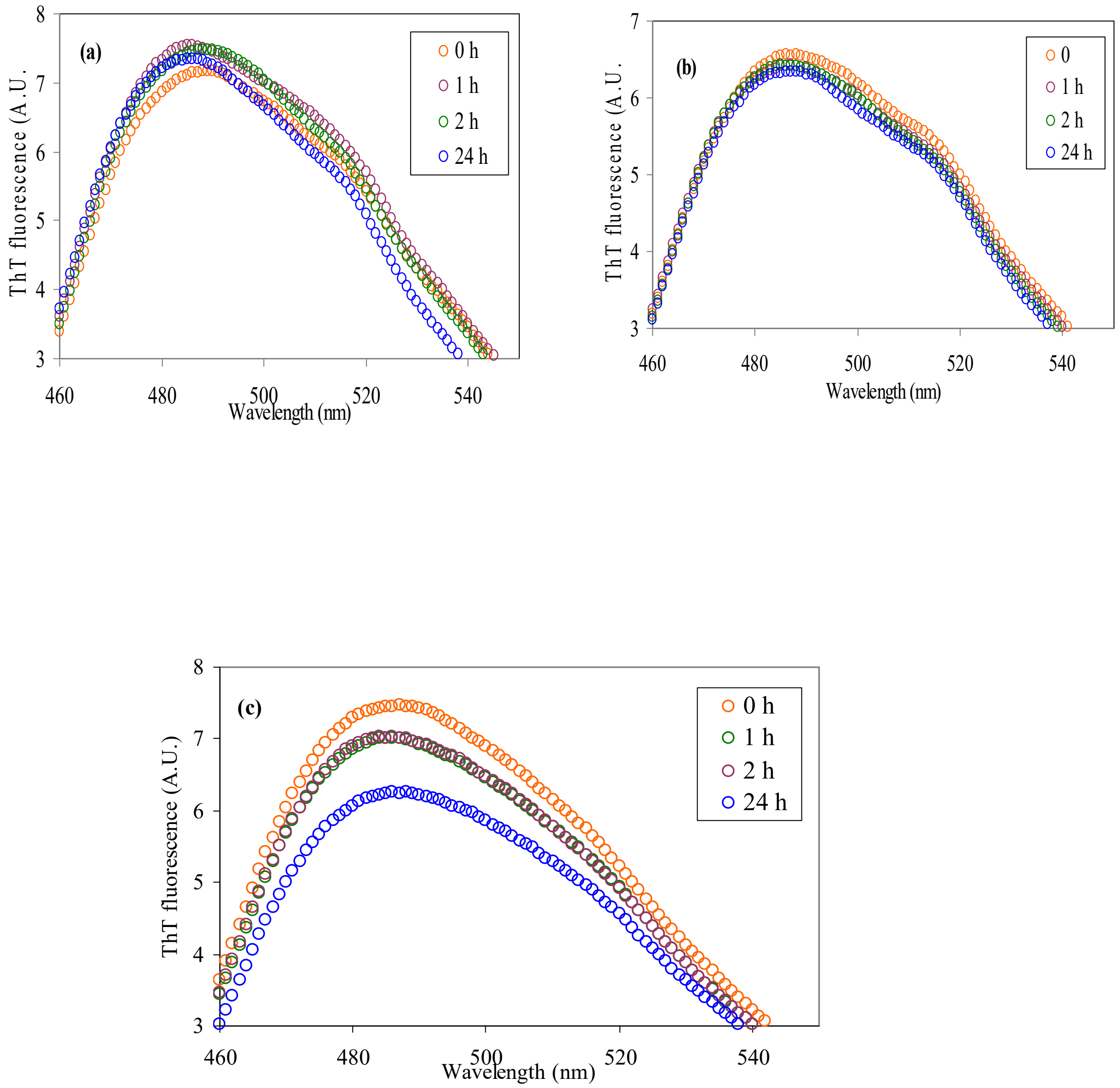

Fig. S5. Effect of different AuNSs; (a) AuNS5, (b) AuNS50 and (c) AuNS100 on the fibrillation of Aβ42 as observed from ThT fluorescence at different time intervals. Samples were excited at 440 nm in 20 mM sodium phosphate buffer (pH 7.2).

(a)

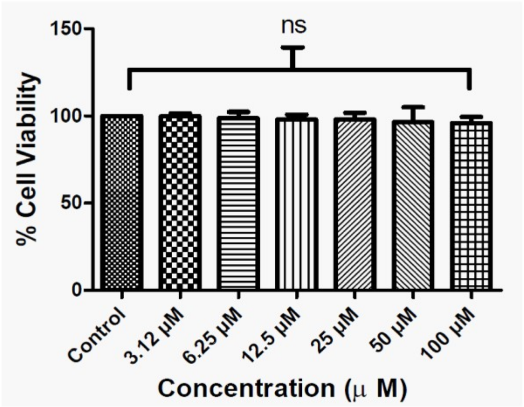

(b)

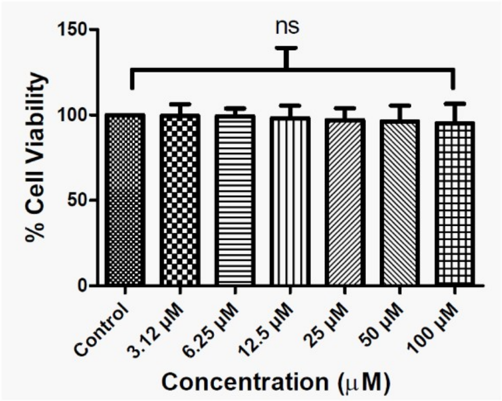

(c)

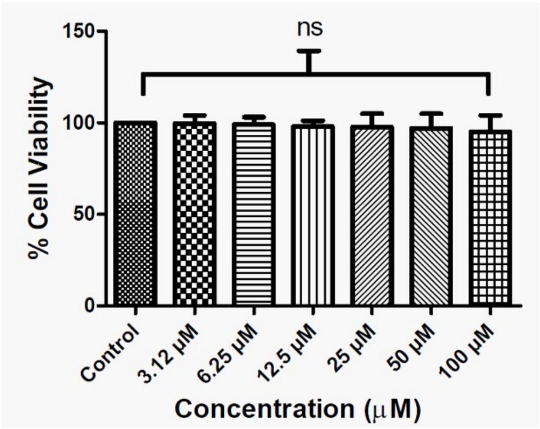

(d)

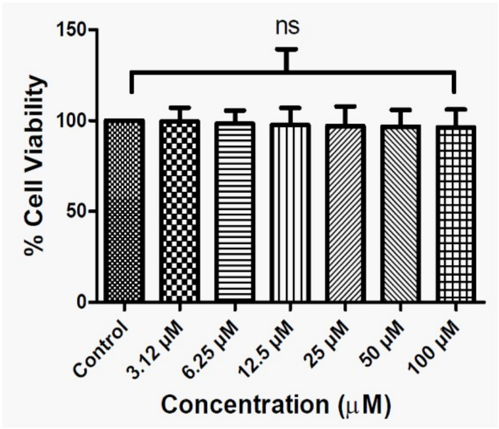

Fig. S6. Cytotoxicity assay using different concentrations of (a) PIMT, (b) AuNS5, (c) AuNS50 and (d) AuNS100 in PC12 derived neurons. Error bars correspond to standard deviations of the value. Data were analyzed using 1-way ANOVA (ns = non-significant, n=4).
